# Diurnal variation of cardiac autonomic activity in adolescent non-suicidal self-injury

**DOI:** 10.1007/s00406-023-01574-1

**Published:** 2023-03-05

**Authors:** Christine Sigrist, Hannah Jakob, Christoph J. Beeretz, Stefanie J. Schmidt, Michael Kaess, Julian Koenig

**Affiliations:** 1 Faculty of Medicine, Clinic and Policlinic for Child and Adolescent Psychiatry, Psychosomatics, and Psychotherapy, University of Cologne, University Hospital Cologne, Cologne, Germany; 2 Clinic for Child and Adolescent Psychiatry, Centre for Psychosocial Medicine, Heidelberg University, Heidelberg, Germany; 3 Department of Clinical Psychology and Psychotherapy, University of Bern, Bern, Switzerland; 4 University Hospital of Child and Adolescent Psychiatry and Psychotherapy, University of Bern, Bern, Switzerland

**Keywords:** Non-suicidal self-injury, Stress regulation, Emotion regulation, Cardiac autonomic activity, Diurnal variation, Developmental psychopathology

## Abstract

Heart rate (HR) and vagally mediated heart rate variability (HRV) are two distinct biomarkers of cardiac autonomic activity. Decreased cardiac vagal activity (or decreased HRV) in particular has been linked with impairments in the functional flexibility of the central autonomic network (CAN), resulting in impaired stress and emotion regulatory capacities. Decreased HRV is widely used as trait marker of psychopathology. Repetitive engagement in non-suicidal self-injury (NSSI) in adolescence correlates with both deficits in stress and emotion regulation, as well as decreased HRV. Existing research has, however, focused on short-term recordings of HR and HRV under resting and phasic conditions. In this study, we examined whether diurnal variation of cardiac autonomic activity, indexed by cosinor parameters of HR and HRV derived from 48 h of ambulatory ECG recording under natural conditions over a weekend, are altered in female adolescents with NSSI disorder compared to controls (HC; *N* = 30 per study group). Several important confounds, including physical activity, were controlled for. Female adolescents with NSSI show higher rhythm-adjusted 24 h mean levels and greater respective amplitude of HR, as well as lower rhythm-adjusted 24 h mean levels and smaller respective amplitude of HRV. Peak levels in both HR and HRV in the NSSI group were reached approximately 1 h later compared to HC. Severity of exposure to early life maltreatment might be linked with altered amplitudes of 24 h HR and HRV. Diurnal rhythms of cardiac autonomic activity might hold promise as objective indicators of disordered stress and emotion regulation in developmental psychopathology, and as such should be investigated in future studies with rigorous assessment and control of potential confounds.

**Supplementary Information:**

The online version contains supplementary material available at 10.1007/s00406-023-01574-1.

## Introduction

Non-suicidal self-injury (NSSI) is the deliberate and self-inflicted damage to body tissue in the absence of suicidal intent [46]. NSSI is fairly prevalent among adolescents and young adults, as estimates suggest that among non-clinical populations, 17% of adolescents and 13% of young adults will have engaged in NSSI at least once in their lifetime [106]. The most important risk factors of NSSI as identified in a recent meta-analysis include mental disorders, low health literacy, early life maltreatment (ELM), bullying, problem behaviors, female gender, and physical symptoms [120]. NSSI is particularly often seen in the context of psychopathological conditions strongly characterized by impaired stress and emotion regulation, most prominently major depressive (MDD) and borderline personality disorder (BPD). Among adolescent psychiatric inpatients, 50 to 80% fulfill the clinical diagnostic criteria for NSSI disorder according to DSM-5 [91, 126], including repetitive engagement (on more than 5 days) in NSSI over the past year [3]. NSSI is linked with other health-risk behaviors, and critically, presents one of the strongest transitional predictors of suicide attempts among adolescents [5, 81, 125]. Concerningly, the rates of both the prevalence of NSSI as well as attempted and completed suicide among adolescents and young adults have shown sharp increases in more recent years [34, 84]. A better understanding of NSSI, not only with regard to psychological and etiological aspects, but also in terms of its neurobiology, is thus of utmost importance.

As noted in a recent review, NSSI is a complex behavior that emerges at the intersection of social, psychological, and biological mechanisms [57]. The social and psychological aspects that contribute to the risk for NSSI are relatively well understood (see e.g. [16, 120], and have guided the development of important and effective psychosocial treatments (see e.g. [66]). In contrast, the biological aspects of NSSI have just begun to come to light (Ref. [39], also see Kaess et al. [57] for a review). Further research aiming to advance the understanding of biological alterations in NSSI is thus indicated.

One aspect that has gained traction as a proposed neurobiological or physiological mechanism involved in NSSI are alterations in the activity and functional flexibility of the autonomic nervous system (ANS). As pointed out by others (e.g. [38]), the *Neurovisceral Integration Model* (*NIM*, Refs. [109, 110]) proposes that physiological, emotion, and cognitive regulation processes are related to each other in the service of goal-directed behavior, as well as adaptability to changing environmental demands [109, 110]—and that the interplay between these functions can contribute to individual differences in mental and physical health and disease. The model further summarizes the relationship between the central nervous system (CNS) and the autonomous nervous system (ANS), and proposes a common cortico-subcortical neural circuit that serves as the structural link between these regulation processes. A network of neural structures [109, 110, 113], amongst others of prefrontal areas, including ventromedial prefrontal cortex and anterior cingulate cortex, and subcortical areas such as the hypothalamus and amygdala, are together called the central autonomic network (CAN). This central autonomic network regulates the ANS through sympathetic and vagal branches that innervate the heart [4, 10]. The dynamic balance between the sympathetic and parasympathetic branches allows for flexible control over the response of the body (e.g. heart) to a range of external and internal stimuli. Importantly, the parasympathetic system is more dominant in maintaining resting heart rate, whereas sympathetic influence on heart rate unfolds in a relatively slower manner, parasympathetic regulation of the heart is much faster, allowing for momentary modulation of cardiac activity [92]. Heart rate variability (HRV), a biomarker that can be derived from heart rate recordings, is the variation in time intervals between heart beats and provides an index of this parasympathetic influence on the heart. Here, as the vagus nerve is the primary parasympathetic nerve [15], when we refer to HRV, we always refer to vagally mediated HRV.

Difficulties in stress and emotion regulation present a central dysfunctional component that is shared among clinical and non-clinical populations engaging in NSSI [2], besides a psychological level [11], this could also be visible at the level of decreased autonomic vagal activity and flexibility [109, 110]. Decreased HRV has been interpreted as objective and transdiagnostic indicator of emotional dysregulation and psychopathology in numerous studies [7, 109, 110]. Meta-analytic studies imply reduced resting-state (short-term) HRV in both adult [59] and adolescent MDD [63, 64], as well as adult [63, 64] and adolescent BPD [122]. Studies on autonomic vagal (parasympathetic) activity in NSSI have shown reduced resting-state HRV and increased HRV reactivity during stress paradigms, such as in response to negative mood induction, in para-suicidal adolescents [23]. In adolescents engaging in NSSI, resting-state short-term HRV is inversely related with the severity of BPD symptomatology, providing evidence for generally altered cardiac autonomic vagal activity in adolescents engaging in NSSI. A small number of studies have also considered short-term HR in individuals engaging in NSSI compared with healthy controls, both at rest and in response to stress, reporting similar results of potentially altered cardiac autonomic activity (e.g. [14, 53]). Although there is substantial interest in autonomic vagal activity in association with emotional dysregulation, evidence currently is limited, and further studies focusing on the various components of ANS activity in NSSI are thus warranted.

In recent years, in addition to the investigation of short-term tonic (resting-state) and phasic (reactivity and recovery) levels of HR and HRV, increasing interest in diurnal components of cardiac autonomic activity has emerged in the context of psychological processes and psychiatric symptoms and disorders [48]. In line with other physiological mechanisms, such as control of core body temperature, blood pressure, or urine volume, cardiac autonomic activity is following a variational pattern with a frequency of an approximate solar day (i.e. 24 h). Diurnal variation of cardiac autonomic activity indexed by HR and HRV, with respective minimum and peak levels during nighttime [44, 47, 49, 74], respectively, can be observed in children from 1 year of age [83, 121], and might undergo marked developmental changes over the lifespan [78]—including diminishing in older age [112]. Research focusing on diurnal rhythms of cardiac autonomic activity in association with psychiatric symptoms and disorders is still scarce, and thus far, focus has been laid on adult populations and non-human primates [47, 49]. Respective studies, however, substantiated sex-specific alterations of diurnal rhythms of HRV in association with depressive symptoms in adult general population samples [19, 32, 111, 115], and altered rhythms of different markers of cardiac autonomic activity have been found in association with depressive symptoms and difficulties in emotion regulation in the context of adult BPD [118].

The findings of altered diurnal rhythms of cardiac autonomic activity in association with certain psychiatric conditions somewhat align with findings from the field of chronobiology, suggesting that circadian rhythms of peripheral physiology, e.g. including body temperature, blood pressure, glucocorticoid secretion, or immune responses, are altered in association with psychopathology ([71, 114], [21]. Growing evidence from both pre-clinical and human studies furthermore substantiates altered circadian rhythms, from molecular genetic up to behavioral levels, in the presence of many psychiatric disorders [61, 119]. Of note, it has been emphasized previously that research should examine disturbances within different components of the circadian system in association with NSSI [123], to date, however, research on this aspect of NSSI disorder is still essentially lacking.

In the present exploratory study, we examined diurnal variation patterns of cardiac autonomic activity in female adolescents with NSSI, assuming to find this functional component of ANS activity to be altered in this vulnerable subpopulation. We were primarily interested in potential alterations of diurnal variation patterns of cardiac autonomic activity in the presence of NSSI disorder, and thus assessed diurnal variation patterns of cardiac autonomic activity in a sample of female adolescents fulfilling DSM-5 criteria of NSSI disorder in comparison to healthy, age-matched control females. We assumed that diurnal tendencies would maximally express themselves only under conditions which sleep is less constrained by external factors (such as, e.g. school commitments) [45], and thus assessed cardiac autonomic activity over two consecutive days on a weekend—as opposed to normal school or working days—and under natural conditions. Based on the strong associations of NSSI with emotional dysregulation, with both MDD and BPD, and with ELM exposure, as well as based on findings of reduced 24-h HRV in association with greater difficulties in emotion regulation in adult BPD [118] and of blunted HRV increase at nighttime in association with both acute and chronic stress exposure [50, 58, 112], we hypothesized that: in female adolescent NSSI disorder, we would find altered diurnal variation patterns of cardiac autonomic activity characterized by reduced rhythm-adjusted mean level and amplitude of HRV, and elevations in respective parameters of HR, as well as potential phase shifts in both measures—compared to healthy, age-matched control females (H1). Furthermore, we examined diurnal variation of cardiac autonomic activity in association with dimensional clinical variables. In these secondary exploratory analyses, we assumed to find significant associations of diurnal parameters of HR and HRV with severity of BPD symptomatology (H2a), as well as the severity of ELM exposure (H2b), depressive symptomatology (H2c), and difficulties in emotion regulation (H2d).

## Methods

### Participants

The present study was conducted at the Department of Child and Adolescent Psychiatry, Heidelberg University, Germany. The present sample comprised *N* = 60 female adolescents aged 12–17 years, half of which fulfilled the diagnostic criteria for NSSI disorder (NSSI group; *N* = 30), while the other half were healthy controls (HC; *N* = 30). Inclusion criteria for the NSSI study group were reporting five or more incidences of self-harm during the past year, and one incidence or more during the past month (also see NSSI disorder criteria; Ref. [3]). Participants in the control group had no lifetime history of self-harm or suicidal behavior, were free from any current psychiatric disorder, and did not receive any psychiatric or psychotherapeutic treatment during the previous 2 years. Participants in the NSSI group were recruited from the outpatient clinic for risk-taking and self-harming behavior (AtR!Sk,*Ambulanz für Risikoverhaltensweisen und Selbstschädigung*; [55]. Healthy controls were recruited from the general community by Email advertisements distributed via Department and University mailing lists. Before study inclusion, all participants underwent extensive clinical interviews to assert eligibility. Individuals with neurological or endocrinological disorders, acute psychotic symptoms, acute suicidality, or lack of understanding of the German language were excluded from the study. All study procedures were approved by the ethics committee of the Medical Faculty at Heidelberg University (Approval Number: S-448/2014) and complied with the Helsinki Declaration of 1975, as revised in 2013. All participants and their legal guardians signed written informed consent prior to participation in the study.

### Measures

#### Clinical assessment

All participants underwent structured clinical interviews and completed self-report measures for further neuro-psychiatric characterization. All assessed measures are detailed in the following. The German version of the *Mini-International Neuropsychiatric Interview for Children and Adolescents* (M.I.N.I.-KID 6.0; Ref. [101], a short and structured diagnostic interview for DSM-IV and ICD-10 psychiatric disorders in children and adolescents, was used to screen for comorbidity in psychiatric diagnoses. The M.I.N.I.-KID has been demonstrated to generate reliable and valid psychiatric diagnoses [101]. To measure NSSI, the German version of the *Self-Injurious Thoughts and Behavior Interview* (SITBI-G, Ref. [26], a clinician-administered interview that assesses suicidal thoughts, suicidal behaviors and NSSI, was used. In prior work, the SITBI has shown strong inter-rater (average *k* = 0.99, *r* = 1.0) and test–retest reliability (average *k* = 0.70, *ICC* = 0.44) over a 6-month period [88]. ELM exposure was measured using the German version of the *Childhood Experience of Care and Abuse Questionnaire* (CECA.Q, Refs. [13, 52]). The CECA.Q assesses four dimensions of ELM, including parental care (neglect and antipathy), parental physical abuse, and sexual abuse (by any adult) before age 17, and has been confirmed a reliable and valid measure to screen for experiences of severe adversity in childhood in clinical [103] as well as community samples [13]. For the present analyses, a binary score was created, indicating presence versus absence of any of the ELM subtypes assessed based on pre-defined cut-offs (i.e. maternal neglect: ≥ 25, paternal neglect: ≥ 24, maternal antipathy: ≥ 25, paternal antipathy: ≥ 25, physical abuse: ≥ 1, sexual abuse: ≥ 1), and a severity score was calculated for each subtype, and overall (for further details on the calculation of respective scores, see [13]). To assess BPD pathology, we used the German version of the *Structured Clinical Interview for DSM-IV-Axis II* (SKID-II, Ref. [31], which has been developed to assess DSM-IV-TR Personality Disorders and has been shown to reliably assess personality disorders in adolescents [98]. For the purpose of the present study, the SKID-II module designed to assess BPD traits was conducted. To assess the current severity of depressive symptoms, the German Version of the *Children’s Depression Inventory* (CDI, Refs. [67, 68] was used ("*Depressionsinventar für Kinder und Jugendliche*", DIKJ, Ref. [105]. The DIKJ is a 26-item self-rating instrument which has been demonstrated to have high internal consistency (*α* = 0.82–0.88), and has widely been used to assess depressive symptomatology in children and adolescents from 8 to 17 years of age [30]. The German version of the *Difficulties in Emotion Regulation Scale* (DERS, Ref. [36]) was used to measure emotional dysregulation. The DERS is a self-report measure designed to assess six dimensions of emotion dysregulation. Subscale scores reflecting each of these factors are available, while the DERS is often reported as a total score. The DERS is used in nearly all studies of trait-level emotion dysregulation in self-harm, but has also widely been used to assess emotion dysregulation in other psychological disorders [28, 29, 40, 124]. The DERS has high internal consistency, good test–retest reliability, and adequate construct and predictive validity [36].

#### Heart rate data processing and analysis

We report recording and analyses of cardiac autonomic data in accordance with the ‘Guidelines for Reporting Articles on Psychiatry and Heart rate variability’ (GRAPH; Ref. [94]). Heart rate was continuously recorded as beat-to-beat intervals at 1024 Hz for 48 h using an ECG Move III chest belt (movisens, Karlsruhe, Germany). The ECG Move III is a state-of-the-art sensor to record ECG at a high-frequency sampling rate through an ambulatory sensor attached to an elastic chest belt, and allows to simultaneously record 3D acceleration and temperature. Participants were instructed to wear the HR recorder for 2 consecutive days over a weekend, starting Saturday morning (10:00 h) until Monday morning (10:00 h), resulting in 48 h of total recording time. Raw ECG recordings were manually visually inspected and preprocessed using UnisenseViewer (FZI Forschungszentrum Informatik and movisens GmbH) and the Kubios Premium 3.0 Software (Kubios, Finland, Ref.[107] allowing for R-Peak detection and correction of raw ECG data. Peak detection was manually corrected and remaining artifacts were removed. Smoothing priors were selected as detrending method (λ 500) for IBI data. Kubios output was saved in the TXT format for later automated readout of corrected inter-beat intervals (IBIs) and analysis of HRV in R [82]. IBIs corresponding to a mean HR < 30 or > 200 bpm were discarded. After pre-processing, each 24 h recording period was segmented into 5-min intervals as available, resulting in a maximum total of 288 segments per measurement day. Segments with less than 30 s of consecutive IBI data were discarded from analyses. For each of the remaining 5 min segments, the following cardiac autonomic parameters were calculated: Mean HR in beats per minute (bpm), and the time-domain measure root mean square of successive inter-beat intervals (RMSSD) in milliseconds (ms) obtained by calculating each successive time difference between heartbeats in milliseconds after which each of the values was squared and the result averaged before the square root of the total was obtained. RMSSD was chosen as HRV metric as it presents a stable and valid measure of autonomic vagal (parasympathetic) activity, and is assumed to be less affected by respiration and artifacts from body movement than other HRV measures [42, 70, 90]—it must be noted, however, that there is no general consensus on this in the field. Based on the 5-min segments available per participant, measures of diurnal rhythmicity of HR and HRV were calculated. In accordance with available guidelines [22, 96] and previous studies focusing on HRV, we applied the cosinor method to quantify diurnal rhythmicity of cardiac autonomic activity. This method, presenting with a least squares approach to time series, has the advantage of being relatively robust in the case of missing and non-equidistant data points [22]. Three individual-level cosine function parameters were estimated for each outcome (HR and HRV) to quantify the diurnal variation pattern: (i) MESOR (Midline Estimating Statistic Of Rhythm) defined as the rhythm-adjusted 24 h mean fitted to the data, (ii) amplitude, defined as the distance between MESOR and the maximum of the cosine curve (i.e. half the extent of rhythmic change in a cycle), and (iii) acrophase, which can be defined as the lag from a defined reference time point (e.g. local midnight when the fitted period is 24 h) of the crest time in the cosine curve fitted to the data [25]. The period was assumed to be 24 h. In sensitivity analyses, population-mean cosinor per study group and day of measurement was calculated, and cosinor model fit was examined for each group. We used the R-package *cosinor2* [86] presenting an extension of the R-package *cosinor* [97] for all cosinor analyses.

#### Covariates

We assessed and calculated potential covariates of cardiac autonomic activity as measured, including age, body mass index (BMI; height/ weight^2^), physical activity, and quality of ambulatory ECG recordings; in addition, sleep duration was calculated to examine potential group differences and bivariate correlations. Of note, when examining HRV measures as outcome of interest, and in association with, e.g. clinical variables, accounting for influential factors known to affect measures of ANS activity is considered to be of high importance (see e.g. [93, 94, 108] for respective guidelines). However, as has been pointed out previously (e.g. [116], in many studies focusing on HRV, and especially in studies of ambulatory ECG measurements, potentially influential factors remain critically unaddressed. In particular, most existing ambulatory HRV studies refrained from considering physical activity as a potential confounder [116], despite evidently strong influences of physical activity levels on HRV. Furthermore, in clinical studies examining associations of psychological variables with ANS functioning, aspects regarding the quality of cardiac data recordings are often not considered. In the present study, we, therefore, aimed to explicitly address the robustness of clinical predictors of interest against these critical factors. To obtain a measure of physical activity, raw acceleration data (measured in *g*) available from ECG Move III sensor recordings was averaged over periods of 60 s, and based on these data, individual step count (total number of steps per day) was calculated using the Movisens Data-Analyzer software (Version 1.13.5, movisens GmbH, Karlsruhe, Germany). From raw acceleration data and using the same software, we also derived sleep duration, wake period, and sensor non-wear time per participant and measurement day. The total number of 5 min segments, calculated as the sum of 5 min segments available per participant over the 48 h of cardiac recording, was used as a proxy of cardiac data quality.

### Statistical procedure

First, data were explored visually (i.e. boxplots, histograms), and univariate normality of continuous data was examined using skewness and kurtosis tests for normality. Descriptive statistics of demographic, physical, and clinical data were calculated, and differences between the two study groups (NSSI group and HC) were examined using independent group *t*-test (or a non-parametric equivalent) for continuous, and χ^2^ test (or Fisher’s exact test in the case of expected frequencies of 5 or less) for categorical data. To test the hypothesis of (H1) group differences in diurnal variation of cardiac autonomic activity between NSSI group and HC, differential rhythmicity analysis was performed, including analysis of significant differences in amplitude and analysis of acrophase shift. Then, we applied univariate multiple linear regression models, controlling for confounders (age, BMI, physical activity, and data quality) thus considering each of the three cosinor parameters of HR and HRV separately to examine potential group differences. Next, bivariate correlations of MESOR, amplitude, and acrophase of HR and HRV with each dimensional clinical predictor of interest (H2a–H2d; i.e. severity of ELM exposure, BPD symptomatology, depressive symptoms, and emotional dysregulation) were explored using non-parametric Spearman’s rank correlation coefficient (Spearman’s *ρ*). Finally, we calculated multivariate single (unadjusted) and multiple (adjusted) linear regression models, allowing us to examine the impact of study group as well as of each dimensional predictor of interest (H1–H2d) on diurnal variation patterns while considering MESOR, amplitude, and acrophase simultaneously as dependent variables in each model. Of note, in these analyses, when examining BPD symptomatology as potential clinical predictor, only the NSSI group was considered; for all remaining clinical predictors, the total sample was considered in respective analyses.

It has to be noted that the present analysis strategy lead to a multiple testing situation where statistically significant differences could emerge by chance, and this needs to be taken into account when interpreting the presented results. Yet, given the exploratory nature of the present analyses, we refrained from p-value corrections for multiple comparisons, given that a strict adjustment for multiple comparisons seems less appropriate in such scenarios.

Data pre-processing as well as descriptive and statistical analyses were performed using Stata/SE (Version 16.0; StataCorp LP, College Station, TX, US), with alpha set to 0.05 (two-sided). Figures were prepared using R version 4.0.2 [95].

## Results

### Descriptive statistics

Descriptive statistics of demographic, physical, and clinical data, separated by study group, are presented in Table 1 below. Mean age (*SD*) of the total sample (*N* = 60) was 14.93 (1.31; range 12–17). No significant differences between study groups regarding age, height, weight, BMI, number of steps per day and time spent asleep were observed. A statistically significant difference in sensor non-wear time was detected, such that the NSSI group on average was characterized by a slightly longer sensor non-wear time (i.e. a higher amount of missing 5-min ECG data segments) compared to HC (see Table 1 below). Regarding clinical psychiatric characteristics, the NSSI group scored significantly higher on the DIKJ, indicating more severe depressive symptoms, compared to HC. Within the NSSI group, NSSI frequency during the past 12 months varied between 5 and 365 days (*M* = 92.17, *SD* = 100.62), and 12 participants (40%) fulfilled diagnostic criteria for BPD (5 or more BPD symptom criteria fulfilled), while 8 participants met criteria for a subthreshold diagnosis (fulfilling 3–4 BPD symptom criteria), and 10 participants were below subthreshold. No participant within the HC group fulfilled more than 1 BPD symptom criterion. As expected, the NSSI group reported ELM exposure to be significantly more severe, and scored significantly higher on all subscales of the DERS as indicative of greater difficulties in emotion regulation, compared to HC.

**Table 1:** Descriptive statistics of demographic, physical, and clinical data, separated by study group

		NSSI (*N* = 30)			Controls (*N* = 30)		NSSI vs. Controls			
		M (SD)	Min–Max		M (SD)	Min–Max	*t* (DF)	*z*	*χ*^2^ (DF)	*p*-value
Age [years]		15.10 (1.06)	13–17		14.77 (1.52)	12–17	0.983 (58)	–		0.330
Height [cm]		165.37 (5.70)	156–175		165.07 (6.03)	150–176	0.198 (58)	–		0.844
Weight [kg]		60.83 (15.42)	38–110		54.60 (6.28)	44–69	–	1.490		0.138
BMI [m/ kg^2^]		22.12 (4.94)	14.69–38.51		20.02 (1.86)	17.18–25.39		1.745		0.081
Step count [No. steps]		11,887 (7135.69)	359–36′609		10′226.62 (4′694.02)	4′671–25′390		0.956		0.346
Sleep time [hours]		7.31 (3.25)	0–16.78		7.64 (1.73)	4.62–11.92		0.237		0.815
Segments [*N* available]		428.31 (154.14)	69–576		510 (98.41)	193–574		-2.78		**0.005**
Non-wear time [min]		5.14 (2.03)	0–8		2.76 (1.53)	0–5		4.90		** <0 .001**

	NSSI (*N* = 23)			Controls (*N* = 30)			NSSI vs. controls			
	*N* (%)	M (SD)	Min–Max	*N* (%)	M (SD)	Min–Max		*z*		*p*-value
CECA.Q^1^										
Total										
Any ELM subtype	17 (73.91)			1 (3.33)						
ELM severity		11.24 (4.03)	5.43–17.57		5.98 (1.26)	4.57–8.86		4.67		** < 0.001**
Subtypes										
Maternal antipathy	8 (34.78)	19.61 (7.76)	8–32	0 (0)	10.5 (2.90)	8–19		4.38		** < 0.001**
Maternal neglect	7 (30.43)	16.91 (7.37)	8–34	0 (0)	10.03 (2.37)	8–17		3.99		** < 0.001**
Maternal physical abuse	2 (8.70)	0.17 (0.65)	0–3	0 (0)	0 (0)	-		1.67		0.348
Paternal antipathy	8 (34.78)	20.57 (9.81)	8–38	0 (0)	10.37	8–19		4.10		** < 0.001**
Paternal neglect	9 (39.13)	19.78 (9.39)	8–40	0 (0)	10.90 (2.72)	8–19		4.07		** < 0.001**
Paternal physical abuse	1 (4.35)	0.04 (0.21)	0–1	1 (3.33)	.07 (0.37)	0–2		0.20		0.846
Sexual abuse	8 (34.78)	1.61 (2.41)	0–7	0 (0)	0 (0)	–		3.54		** < 0.001**

	NSSI (*N* = 30)			Controls (*N* = 30)			NSSI vs. Controls		
	M (SD)		Min–Max		M (SD)	Min–Max		*z*	*p*-value
DERS									
Total score	111.76 (23.36)		59–149		54.30 (10.72)	36–82		6.427	** < 0.001**
Non-acceptance	17.37 (5.81)		6–27		9.63 (3.52)	6–18		4.869	** < 0.001**
Goals	17.50 (4.64)		8–24		9.33 (3.97)	5–20		5.387	** < 0.001**
Impulse	17.13 (6.67)		6–29		8.37 (2.98)	6–17		5.158	** < 0.001**
Awareness	20.9 (5.23)		9–29		11.03 (3.55)	7–21		5.629	** < 0.001**
Strategies	27.37 (6.83)		14–39		11.07 (3.71)	8–23		6.373	** < 0.001**
Clarity	17.20 (5.01)		7–25		7.60 (2.25)	5–12		6.039	** < 0.001**

	NSSI (N = 30)			Controls (N = 30)					
	M (SD)		Min–Max	*N* (%)	M (SD)	Min–Max			
NSSI frequency									
Past 6 months [days]	47.37 (43.37)		0–120		-	-			
Past 12 months [days]	92.17 (100.62)		5–365		–	–			

	NSSI (*N* = 30)			Controls (*N* = 30)			NSSI vs. Controls		
	*N* (%)	M (SD)	Min–Max	*N* (%)	M (SD)	Min–Max		*χ*^2^ (DF)	*p*-value
BPD									
Full diagnosis	12 (40.00)	6 (0.73)	5–8	0	–	–		30.00 (2)	** < 0.001**
Sub-threshold	8 (26.67)	3.63 (0.52)	3–4	0	–	–			
Below subthreshold	10 (33.33)	1.5 (0.53)	1–2	30 (100.00)	0.07 (0.25)	0–1			

	NSSI (*N* = 30)			Controls (*N* = 30)			NSSI vs. Controls		
		M (SD)	Min–Max	*N* (%)	M (SD)	Min–Max		*t* (DF)	*p*-value
DIKJ									
Total score		25.52 (10.65)	3–44	30	5.27 (3.04)	0–13		9.95 (53)	** < 0.001**

	NSSI (*N* = 30)			Controls (*N* = 30)						
	*N* (%)			*N* (%)						
M.I.N.I.-KID										
ICD-10: F0	2 (8.00)			–						
ICD-10: F1	1 (4.00)			–						
ICD- 10: F2	–			–						
ICD-10: F3	11 (44.00)			–						
ICD-10: F4	3 (12.00)			–						
ICD-10: F5	–			–						
ICD-10: F6	7 (28.00)			–						
ICD-10: F8	–			–						
ICD-10: F9	1 (4.00)			–						

Bold font indicates statistical significance

^1^*N* = 7 missings in the NSSI group

**Fig. 1:**
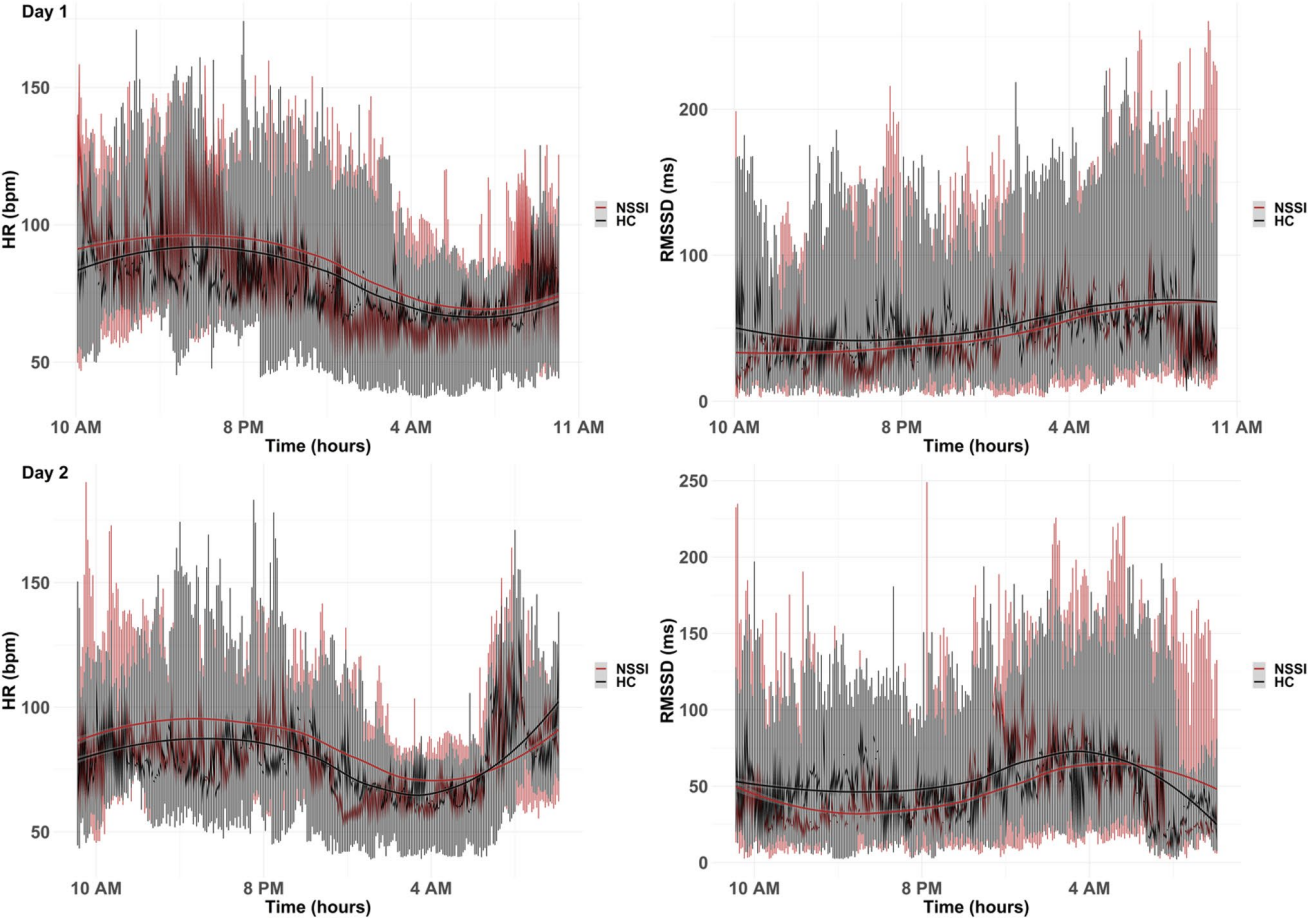
Visualization of 48 h cardiac autonomic data recordings split into 5-min segments of mean HR and HRV (RMSSD), separated by day of measurement and study group

Considering cardiac autonomic data, a total of 27′879 (80.67%) 5-min segments were available for further analysis. Participants were excluded if cosinor parameters could not be fitted reliably based on the data available (see [22] for some guidance). Of note, ECG data were missing for one participant in the NSSI group, due to significant sensor non-wear time. Data from the remaining participants were retained, however, the number of available segments varied significantly between participants as well as between study groups (see Table 1). In Fig. 1, diurnal rhythms of cardiac autonomic data of mean HR (bpm) and HRV (rMSSD, in ms) over 48 h (i.e. 2 measurement days) are shown, including smoothed conditional means to aid visual detection of potential underlying patterns and group differences. Descriptive information on population-mean cosinor parameters for each study group, as well as respective cosinor model fit statistics of group level data, are shown in Table 2*.* Rhythm detection tests suggested that the respective cosinor models fit the present data well on a group level. In Supplementary Table 1 in the online supplement, descriptive statistics of individual-level cosinor parameters used in further statistical analyses, summarized per study group, can be seen.

**Table 2:** Descriptive data of population-mean cosinor parameters (by study group), respective model fit, and group comparison results (differential rhythmicity analyses)

	NSSI			HC			Cosinor model fit^a^				Differential rhythmicity analysis^b^			
							NSSI		HC		NSSI vs. controls			
	*N*	Pop. Mean	Conf. Int	*N*	Pop. mean	Conf. int	F (DF^1^, DF^2^)	*p*-value	F (DF^1^, DF^2^)	*p*-value	*F*(DF^1^, DF^2^)	*t*(DF)	*p*-value	SMD^c^ 95% CI
HR														
MESOR	29	85.98	81.74–90.22	30	79.44	76.14–82.74	39.87 (2, 27)	** < 0.001**	89.85 (2, 28)	** < 0.001**	6.27 (1, 57)		**0.015**	0.64 [0.12, 1.16]
Amplitude	29	12.42	9.26–15.56	30	12.30	10.43–14.17						− 2.19 (57)	**0.032**	
Acrophase	29	1.81	1.63–2.04	30	1.56	1.40–1.71					11.08 (1, 57)		**0.002**	
HRV														
MESOR	29	44.82	37.38–52.26	30	55.79	48.41–63.18	24.99 (2, 28)	** < 0.001**	10.35 (2, 27)	** < 0.001**	4.59 (1, 57)		**0.036**	− 0.56 [− 1.06; − 0.03]
Amplitude	29	13.90	7.68–20.11	30	14.45	10.30–18.60						1.86 (57)	**0.034**	
Acrophase	29	5.02	− 4.75–5.33	30	4.73	4.57–4.90					5.69 (1, 57)		**0.021**	

Bold font indicates statistical significance

*HR* heart rate, *HRV* heart rate variability (RMSSD, ms), *MESOR* midline estimating statistic of rhythm

^a^Based on rhythm detection tests; significant results suggested that the respective cosinor models fit the present data well on a group level

^b^To test the hypothesis of significant group differences in diurnal variation of cardiac autonomic activity between NSSI group and HC, differential rhythmicity analysis was performed using the R-package ‘cosinor 2’ [22, 86, 96], including analysis of significant differences in amplitude and analysis of acrophase shift

^c^Hedge’s *g* reported

### Analyses results

#### Differences in diurnal variation patterns of HR and HRV between study groups

**Fig. 2:**
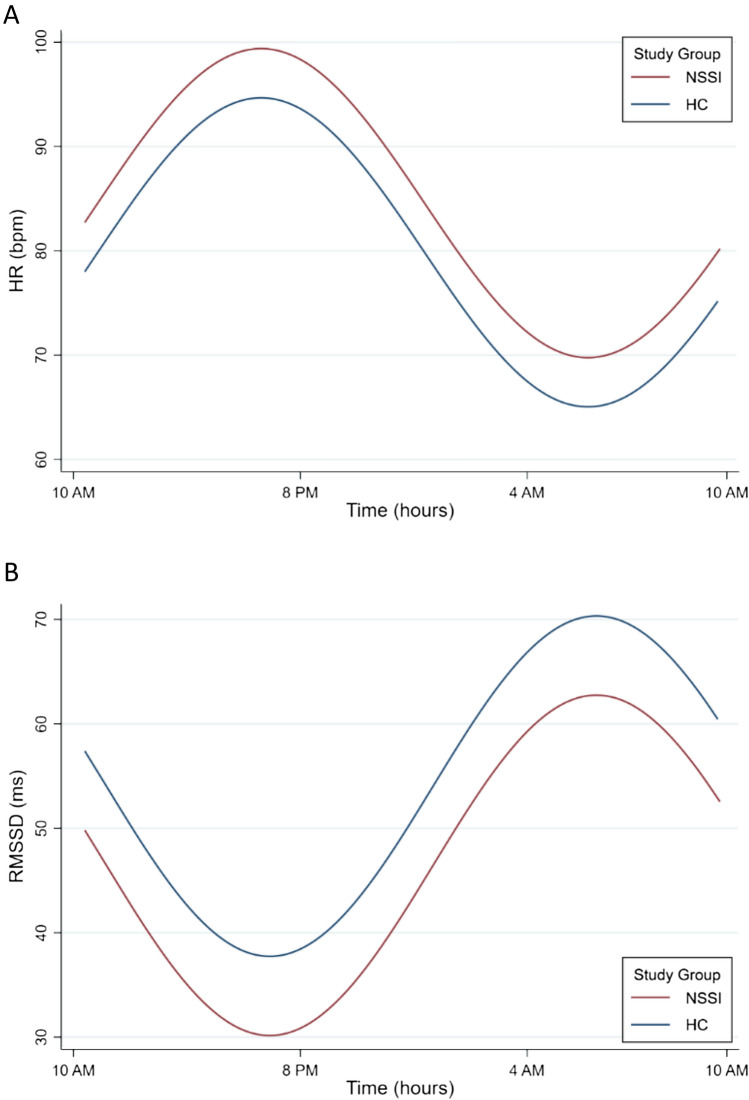
Illustration of the effect of study group on CVP of HR (**A**) and HRV (**B**). Differential rhythmicity analysis yielded statistically significant differences in MESOR, amplitude, and acrophase of both HR and HRV between study groups. Of note, robustness of NSSI disorder as a clinical predictor of CVP against critical confounds was observed for HR, but not HRV

Graphical consideration of cardiac autonomic data implicated potential group effects regarding both 24 h HR and HRV (Fig. 1). Cosinor-specific group comparisons, including differential rhythmicity analysis and analysis of acrophase shift, revealed statistically significant differences between study groups (Table 2). Significant differences in rhythm-adjusted mean levels (MESOR; Fig. 2) considering both HR (*F*_1,57_ = 6.27, *p* = 0.015) and HRV (*F*_1,57_ = 4.59, *p* = 0.036) were observed, as well as significant acrophase shifts (HR: *F*_1,57_ = 11.08, *p* = 0.002; HRV: *F*_1,57_ = 6.27, *p* = 0.015). Of note, since acrophase of both HR and HRV were significantly different between study groups, potential differences in amplitude could not be examined reliably using differential rhythmicity analysis [86]. Group comparisons concerning amplitudes were therefore performed using unpaired group *t*-tests, which yielded statistically significant differences in amplitudes of HR (*t*_57_ = − 2.19, *p* = 0.032) and HRV (*t*_57_ = 1.86; *p* = 0.034) between study groups (Fig. 2). Overall, the NSSI group showed a significantly higher rhythm-adjusted mean HR and lower HR amplitude, as well as significantly lower rhythm-adjusted mean HRV and higher respective amplitude, compared to HC. Furthermore, in the NSSI group compared to HC, the acrophase of HR and HRV was shifted significantly, such that peak levels in both HR (morning peak) and HRV (peak at nighttime) were reached at a later time point in the NSSI group. Considering HR, conversion of respective indices (from radians to clock hours) revealed acrophase was reached approximately 0.96 h (57 min 28 s) later, while concerning HRV, acrophase was shifted for 1.10 h (1 h 5 min 57 s) with clock time in NSSI compared to HC.

Next, we examined whether adjusting for potential confounds (i.e. age, BMI, physical activity, and cardiac data quality indexed by available 5-min segments) using multiple linear regression altered the effect of study group as a statistically significant predictor of cosinor parameters of HR and HRV. In adjusted models, study group was still a statistically significant predictor of the cosinor parameter MESOR of 24 h HR, *B* = − 0.32, t_57_ = 2.28, *p* = 0.027, while BMI, *B* = − 0.42, *t*_57_ = − 0.27, *p* = 0.008, and the quality of cardiac data, *B* = − 0.33, *t*_57_ = − 2.60, *p* = 0.012, significantly influenced MESOR of HR in the respective model. Study group was no longer a statistically significant predictor of MESOR of HRV, or amplitude or acrophase of HRV or HR, in adjusted univariate multiple linear regression models.

#### Correlational analyses

Next, we were interested in examining a range of dimensional clinical variables as predictors of diurnal variation patterns of HR and HRV, either considering the NSSI group in isolation (i.e. severity of BPD symptoms) or the total sample (i.e. severity of ELM exposure, depressive symptoms, and emotional dysregulation). In correlational analyses using Spearman’s *ρ*, we examined bivariate correlations between each of these clinical predictors of interest, as well as further variables and confounds, and the cosinor parameters MESOR, amplitude, and acrophase of HR and HRV. In the Supplementary Figure in the online supplement, a heat map of the corresponding correlation coefficients can be seen. A significant negative correlation was observed between sleep duration and MESOR of HR (Spearman *ρ* = − 0.33, p = 0.010), and MESOR of HR was positively linked with step count (Spearman *ρ* = 0.30, *p* = 0.024). Amplitude of HR was significantly correlated with quality of cardiac data (Spearman *ρ* = 0.36, *p* = 0.005). Regarding HRV, a significant association between amplitude of HRV and step count was identified (Spearman *ρ* = 0.28, *p* = 0.032). No other statistically significant relationships were observed in bivariate correlation analyses.

#### Multivariate linear regression results

In Table 3, full model results from multivariate simple linear regression models are shown.

**Table 3:** Full results from multivariate single linear regression analyses (unadjusted models)

		HR						HRV					
		*N*	*B*	95% CI	Std	*z*	*p*	*N*	*B*	95% CI	Std	*z*	*p*
MESOR	Study group	**59**	**00.62**	**0.14–1.10**	**0.25**	**2.55**	**0.011**	**59**	**− 0.54**	**− 1.03–− 0.05**	**0.25**	**− 2.18**	**0.028**
	BPD symptoms	29	− 0.06	− 0.51–0.40	0.23	− 0.24	0.811	29	0.08	− 0.33–0.49	0.21	0.37	0.710
	ELM severity	59	0.15	− 0.09–0.39	0.12	1.25	0.212	59	0.25	0.01–0.50	0.14	− 1.11	0.268
	Depressive symptoms	59	0.14	− 0.09–0.37	0.12	1.20	0.230	59	− 0.14	− 0.41–0.12	− 13	− 1.06	0.291
	Emotional dysregulation	59	0.19	− 0.06–0.44	0.13	1.49	0.136	59	− 0.15	− 0.40–0.10	0.13	− 1.20	0.230
Amplitude	Study group	**59**	**− 0.55**	**− 1.04–0.07**	**0.25**	**− 2.23**	**0.026**	59	0.47	− 0.02–0.97	0.25	0.34	0.736
	BPD symptoms	29	0.22	− 0.21–0.66	0.22	1.02	0.307	29	− 0.38	− 0.86–0.11	0.25	− 1.52	0.129
	ELM severity	**59**	**− 0.26**	**− 0.49–− 0.04**	**0.12**	**− 2.29**	**0.022**	**59**	**0.25**	**0.01–0.50**	**0.12**	**1.98**	**0.048**
	Depressive symptoms	59	− 0.14	− 0.39–0.11	0.13	− 1.08	0.282	59	0.21	− 0.05–0.47	0.13	1.58	0.114
	Emotional dysregulation	59	− 0.12	− 0.37–0.13	0.13	− 0.92	0.358	59	0.10	− 0.15–0.36	0.13	0.81	0.419
Acrophase	Study group	59	− 0.03	− 0.54–0.47	0.26	− 0.13	0.900	59	0.09	− 0.42–0.59	0.26	0.34	0.736
	BPD symptoms	29	− 0.46	− 0.96–0.04	0.26	− 1.79	0.073	29	0.13	− 0.37–0.63	0.26	0.51	0.613
	ELM severity	59	0.11	− 0.13–0.35	0.12	0.88	0.377	59	0.03	− 0.23–0.30	0.14	0.28	0.783
	Depressive symptoms	59	0.09	− 0.14–0.31	0.12	0.74	0.460	59	− 0.04	− 0.30–0.22	0.13	− 0.31	0.756
	Emotional dysregulation	59	0.01	− 0.25–0.26	0.13	0.03	0.978	59	0.01	− 0.24–0.27	0.13	0.10	0.919

The cosinor parameters MESOR, amplitude, and acrophase of HR and HRV, respectively, were considered as multivariate outcome. Bold font indicates statistical significance

Standardized beta coefficients are reported

*HR* heart rate (bpm), *HRV* vagally mediated Heart rate variability (RMSSD, ms), *MESOR* midline estimating statistic of rhythm

##### Unadjusted multivariate linear regression models

**Fig. 3:**
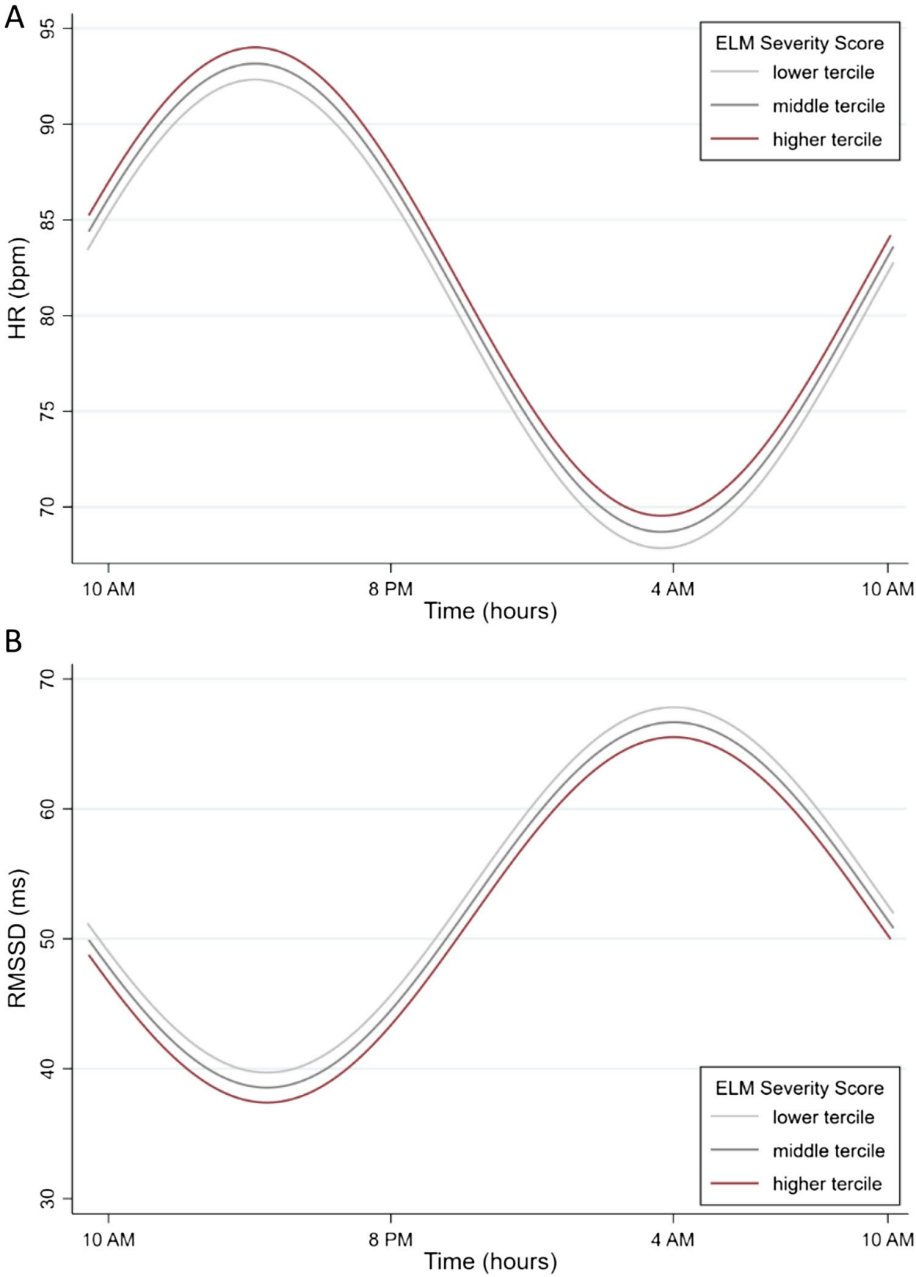
Illustration of the effect of ELM exposure severity on amplitude of HR (**A**) and HRV (**B**), considering both the NSSI study group and HC. Multivariate simple linear regression models suggested a statistically significant influence of the severity of ELM exposure on amplitude of both HR and HRV

In line with the results from simple group comparisons (Sect. “Differences in diurnal variation patterns of HR and HRV between study groups” above and Fig. 2), the variable study group was identified as a significant predictor of both MESOR, *B* = 0.62, SD = 0.25, *z* = 2.55, *p* = 0.011, and amplitude, *B* = − 0.55, SD = 0.25, *z* = − 2.23, *p* = 0.026, of HR, and predicted significant differences in MESOR of HRV (*B* = − 0.54, SD = 0.25, *z* = − 2.18, *p* = 0.028). Severity of ELM exposure was identified as a significant predictor of amplitude of both HR, *B* = − 0.26, SD = 0.12, *z* = − 2.29, *p* = 0.022, and HRV, *B* = 0.25, SD = 0.12, *z* = 1.98, *p* = 0.048 (Fig. 3). None of the remaining clinical predictors was significantly linked with diurnal variation patterns of HR or HRV in unadjusted multivariate regression models.

##### Fully adjusted multivariate linear regression models

**Fig. 4:**
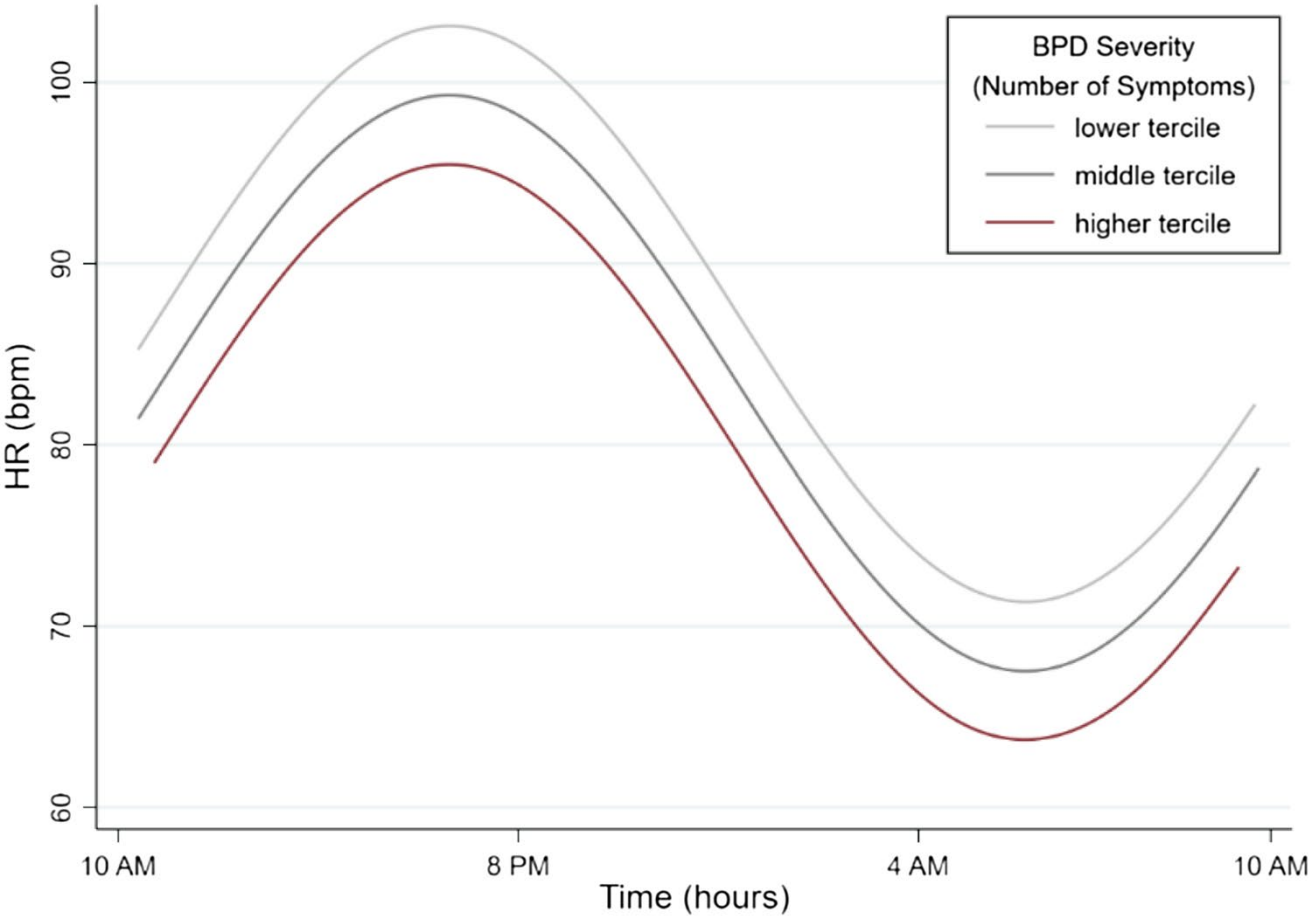
Illustration of the effect of BPD symptomatology on amplitude of HR, considering data from the NSSI study group in the respective analyses. Multivariate multiple linear regression (but not unadjusted) models yielded a statistically significant influence of the severity of BPD symptomatology on CVP of 24 h HR (amplitude)

Results for significant predictors from multivariate multiple linear regression models are presented in Supplementary Table 2 in the online supplement. After including the covariates age, BMI, step count, and cardiac data quality in multivariate linear regression models, in line with univariate multiple regression results (Sect. “Differences in diurnal variation patterns of HR and HRV between study groups” above), the variable study group was still a significant predictor of MESOR of HR (*B* = 0.57, SD = 0.24, *z* = 2.41, *p* = 0.016), while in the respective model, data quality significantly influenced all three cosinor parameters (MESOR, amplitude and acrophase), BMI significantly influenced MESOR, and age the amplitude, of HR (please see *Supplementary Table 2* for detailed reporting of respective statistics). Group differences in MESOR of HRV or amplitude of HR were no longer statistically significant, also in line with univariate linear regression results. In fully adjusted models, the severity of BPD symptomatology (NSSI subgroup) additionally predicted amplitude of HR, *B* = 0.43, SD = 0.19, *z* = 2.26, *p* = 0.024 (Fig. 4 below), while in the respective model, data quality again significantly influenced all three cosinor parameters (MESOR, amplitude and acrophase), BMI significantly influenced MESOR, and age the amplitude, of HR. None of the remaining clinical predictors was significantly linked with diurnal variation patterns of HR or HRV in these models.

## Discussion

In the present study, based on exploratory analyses, we examined diurnal variation patterns of cardiac autonomic activity in female adolescents with repetitive NSSI, and age-matched healthy controls. We hypothesized (H1) that in NSSI disorder, we would find altered diurnal variation patterns of HR and HRV, quantified by using cosinor function parameters on 48 h of preprocessed cardiac autonomic data collected over one weekend, as compared to HC. We also examined diurnal variation patterns of HR and HRV in association with a range of dimensional clinical predictors (H2a–H2d; i.e. severity of BPD symptomatology, ELM exposure, depressive symptoms, and emotional dysregulation) in secondary analyses, and tested the robustness of our results against potential confounders (i.e. age, BMI, step count, and cardiac data quality).

In partial support of our main hypothesis (H1), (unadjusted) differential rhythmicity analyses indeed revealed significant differences in diurnal patterns of cardiac autonomic activity between the two study groups. Within the present recoding period of 48 h, the NSSI group showed significantly higher and lower rhythm-adjusted mean levels of both 24 h HR and HRV, respectively, compared to HC. Furthermore, significant alterations in amplitudes regarding both HR and HRV were observed in the NSSI group: amplitude of HR was significantly higher, while amplitude of HRV was significantly lower, compared to HC. Finally, significant acrophase shifts were observed regarding both HR and HRV, such that the NSSI group reached peak levels in both these cardiac autonomic parameters approximately 1 h later.

The present finding of significantly lower rhythm-adjusted mean levels of HRV and significantly higher rhythm-adjusted mean levels of HR in NSSI compared to HC support the notion of chronically low levels of cardiac vagal (parasympathetic) activity in NSSI relative to HC. This expands on previous findings of lower short-term autonomic vagal activity in individuals with NSSI, indexed by reduced resting-state HRV and increased HRV reactivity (e.g. [23]). Based on the NIM [109, 110], shared neural circuits involved in the regulation of ANS activity and emotion form a functional overlap between autonomic arousal and emotion regulation. In a further elaboration of the NIM with regard to the developmental period (*dynamic model of neurovisceral integration in development*, Ref. [62], it has been suggested that the functional interaction of the ANS and CNS is shaped early in the course of life, and that adolescence might present the most sensitive period in the development of this circuitry [62]. In normative development, vagal influence over cardiac autonomic activity is proposed to increase, indexed by normative decreases in HR and increases in HRV, respectively. ANS maturation, in turn, is assumed to be critical for patterns of PFC maturation and associated regulatory capacities over subcortical regions to emerge, affecting stress and emotion regulation [62]. Chronically low HRV and high HR, respectively, as observed in the present group of female adolescents with NSSI disorder compared to HC, might reflect relative absence of normative ANS maturation. ANS dysmaturation in the NSSI group might further reflect disruption in developmental patterns of PFC maturation (i.e. *cortical thinning*) [62] linked with heightened sensitivity to stressors and maladaptive coping—resulting in an increased risk of psychopathological outcomes in the long run. Critically, though, given the current lack of longitudinal research on the association between ANS maturation and developmental psychopathology [62], no conclusions can currently be drawn as to whether the observed alterations in ANS activity patterns might be causative of NSSI behavior or merely correlational, or whether they may represent systemic adaptations as assumed, e.g. in association with ELM exposure (e.g. [56].

The findings of significantly altered amplitude and significant phase shifts of cardiac autonomic activity in NSSI disorder somewhat align with previous studies in the field of chronobiology, suggesting disturbed circadian patterns in association with psychiatric symptoms and disorders, which are seen already in young age [37, 41, 69, 77, 85, 87]. In chronobiological research, significant changes in amplitude and acrophase (*phase shifts*) of circadian rhythm, commonly referred to as circadian rhythm disruption [61], have been associated with increased risk for physical and psychiatric disorders [8]. Relatedly, circadian disruption in the form of misalignment between the circadian system and daily sleep–wake behaviors were shown to adversely affect mood levels and cortical activity underlying mood regulation [20]. Again, brain imaging studies suggest adolescence to present a sensitive period for brain maturation and particularly maturation of prefrontal regions, where dramatic changes on both structural and functional levels can be observed [17, 33, 62, 89, 104]. Such changes might be linked with lower impulse control inhibition, poorer decision making in emotional context, greater risk-taking behavior, and heightened patterns of emotional instability in adolescence [6, 17]. Provided that adolescence is also characterized by normative changes in the circadian rhythm of numerous physiological processes (e.g. physiologically mediated shifts toward evening preference, which also contribute to irregular sleep schedules and a general mismatch between behaviors and circadian rhythms, Ref. [18], altered amplitudes and significant phase shifts of physiological indicators, as observed in the present sample of female adolescent with NSSI, might indicate even stronger mismatch between different circadian processes and behavior, further indicating heightened vulnerability to stress. While sleep deprivation in adolescents has been associated with deficits in emotion regulation (e.g. [9]), there is substantial evidence that sleep problems such as disrupted sleep, poor sleep quality and shorter sleep duration increases risk of engagement in NSSI—and that such associations are particularly strong among adolescents [60]. Interestingly, studies examining attendances in accident and emergency departments related with NSSI behavior [43], and studies using high‑frequency experience sampling in help-seeking populations engaging in NSSI [65], report that among adolescents, this behavior is observed most frequently in the evening hours, suggesting developmental specificities in diurnal rhythms which might also affect NSSI behavior.

The present results considering dimensional clinical predictors (H2a–H2d) using multivariate regression models suggested ELM exposure (unadjusted analyses) and the severity of BPD symptoms (adjusted) to present significant dimensional predictors of diurnal variation patterns of both HR and HRV. In participants reporting higher severity of ELM exposure, a significantly lower amplitude in HR and respective higher amplitude in HRV was observed. Furthermore, a higher severity of BPD symptomatology (NSSI subgroup) was significantly linked with lower amplitude of HR. Considering the etiology of both NSSI disorder and BPD, exposure to adverse environmental factors during critical developmental periods are considered important mediators of the respective disorder, and exposure to severe forms of early life stress in particular, such as ELM, has strongly and consistently been linked with the emergence of NSSI [27, 35, 54, 76, 80] and BPD [54, 72, 80].

Many neural structures, circuits, and neuro-humoral systems involved in stress and emotion regulation are altered in association with ELM exposure (see, e.g. [12, 24, 102]), and circadian disruption might be a critical patho-mechanism linking ELM exposure with increased risk of psychopathology [61, 119]. Multilevel interactions between the major stress and circadian systems are vital for adaptive functioning of central bio-behavioral mechanisms, while exposure to severe traumatic stress can critically alter the functional interplay between those systems [1]. Altered circadian rhythms, indicating circadian dysregulation after severe stress exposure, may present one of the core features of trauma-related psychopathology, mediating enduring neurobiological correlates of traumatic exposure through maladaptive stress regulation [1]. The present findings of altered diurnal parameters of cardiac autonomic activity in association with chronic severe stress exposure (i.e. ELM) as well as BPD (which by some has been considered a trauma-related disorder) align with these findings. Yet, no conclusions regarding potential alterations of endogenous rhythmicity can be drawn based in the present findings, due to critical limitations. Importantly, given the lack of control over exogenous stress related factors as a potential explanation of the present findings of altered diurnal HR and HRV in the NSSI study group, we are not able to draw any conclusions regarding potential alterations in endogenous circadian rhythms based on our study results. As outlined previously, alterations in ANS activity could present an important risk factor in adolescence for the development of more severe psychopathology. Thus, potential alterations in circadian rhythms of cardiac autonomic activity in NSSI disorder could present interesting targets for future investigation, also given the relative ease and high temporal resolution of ECG measurement. Further insights into circadian rhythms of physiological systems in adolescence in general, and potential alterations among vulnerable subgroups, might be important for the development of novel neurobiological-based interventions that have the potential to restore autonomic vagal activity and affect regulatory processes. Indeed, to our knowledge, no study to date assessed potential alterations in circadian variation patterns of cardiac autonomic activity in adolescent NSSI, nor in association with any other psychiatric disorder or respective symptoms, in childhood and adolescence. Thus, rigorous chronobiological oriented studies are warranted, assessing and examining potential alterations in circadian variation of physiological rhythms in NSSI. Crucially, a better understanding of the mechanisms of how early exposure to severe and chronic stress affects physiological regulatory systems in the long run will thereby continue to present an important research objective, allowing to further advance preventive and interventional strategies in vulnerable risk-populations, including adolescents with NSSI disorder.

While previous studies in adults and non-human primates substantiated potential alterations in diurnal variation of cardiac autonomic activity in association with depressive symptoms and disorders, as well as with difficulties in emotion regulation in the context of BPD [47, 49, 115, 118], in the present study of female adolescents with and without NSSI disorder, we did not find significantly altered diurnal variation of HR or HRV in association with depressive symptoms or emotional dysregulation. Of note, previous studies examining the association of diurnal variation of cardiac autonomic activity with depressive symptoms [47, 115] recruited a different target population of relatively healthy adult individuals. Furthermore, the only study examining diurnal variation of cardiac autonomic activity in clinical populations of psychiatric patients again recruited adults, and furthermore, a different analytical approach had been adopted: While we and others [47] used cosinor to derive parameters of diurnal rhythmicity (as suggested also in existing recommendations, see [48]), in the aforementioned study [118], in the diurnal analysis, ECG recordings were segmented in 30 min epochs and averaged according to the sleep or wake periods, allowing to examine associations of clinical variables with mean level during these two periods, as well as with mean-level differences between sleep and wake periods, while not allowing for inferences regarding the range of oscillation or potential phase shift. Although insightful, the results of previous studies in psychiatric populations might, therefore, not readily be comparable to the findings of the study at hand. Of note, while NSSI disorder might be associated with sleep disruptions [123], in the present study, we did not find statistically significant differences in sleep duration derived from acceleration data between study groups. Indeed, previous studies could show that sleep regularity and timing reflect well-being better than sleep duration [8, 51, 69, 79], and thus, future studies concerned with cardiac autonomic measures and their diurnal variation in association with psychopathology might measure the timing and regularity of sleep rather than mere sleep duration, and explore and also control for associations between sleep and patterns of physiological variation.

The presented results must be considered within the context of critical limitations inherent to the study at hand, which will hopefully inform the conduct of future research. As mentioned above, a major drawback of this study is the lack of inclusion of data on experiences throughout the day. Cardiac autonomic measures fluctuate in response to a host of different factors, and no definite conclusions can be drawn with regard to typical vs. atypical diurnal variation patterns of HR and HRV without further knowledge on internal and environmental influences participants might have been exposed to over the 48 h period of cardiac data recording. Moreover, besides the covariates presently included, there might be a range of further variables that could influence (diurnal) cardiac autonomic activity, including puberty status, menstrual cycle, psychotropic medication, and alcohol, nicotine, or caffeine consumption [73, 75, 94, 99, 99, 100, 100, 117]. Of these, the lack of control for potential medication in the NSSI group presents a particularly severe drawback of this study, and any form of medication should be considered in future studies focusing on HRV in NSSI. Of note, one previous study assessed potential covariates such as consumed units of tobacco, coffee, and alcohol on an hourly basis using EMA [115]. Finally, in the present study, which was largely based on assumptions put forth in the NIM ([109, 112, 113]), and in line with many previous research studies in the field (e.g. [47, 49, 115]), we have focused on a HRV measure of autonomic vagal activity, however, sympathetic- or baroreflex-based HRV measures might provide further valuable insight into potential alterations of cardiac autonomic activity in NSSI, for example, one previous study has investigated a host of different HRV metrics in association with BPD [118]. Thus, future research, when expanding on the present findings, might focus on different HRV measures, to gain further valuable insight. Based on the present limitations, future studies should more rigorously measure and control for potential confounders (e.g. socio-emotional stressors, medication, alcohol and nicotine consumption, etc.), potentially using high-frequency EMA, in concert with recruitment of larger and more diverse samples, and considering a variety of cardiac autonomic measures, to critically extend and further substantiate the validity and credibility of the present results.

## Conclusion

While the investigation of the link between psychiatric symptoms and disorders and diurnal variation patterns of cardiac autonomic activity is an emerging field of interest, to the best of our knowledge, the present study is the first to investigate this link with regard to NSSI disorder in adolescence. Overall, our study revealed a marked difference in diurnal variation patterns in cardiac autonomic activity in female adolescents with NSSI disorder compared to well-matched controls. The present study findings can be seen as first indication of a potential desynchronization of cardiac autonomic activity with environmental rhythms in a potentially stress-sensitive subgroup of female adolescents characterized by repetitive engagement in NSSI. Yet, as mentioned previously, the biological mechanisms underlying NSSI have just begun to come to light [57]. Future studies will have to show whether the here observed diurnal alterations are exogenous or endogenous in nature. The present findings, therefore, have implications for the future conduct of neurobiological-driven research in the field of developmental psychopathology. Moreover, this study highlights critical methodological aspects in the ambulatory collection and analysis of cardiac data in psychiatric samples—which could be addressed thoroughly through refinements of future study protocols.

### Supplementary Information

Below is the link to the electronic supplementary material.

Supplementary file1 (DOCX 164 KB)

## Data Availability

Anonymized data are available upon reasonable requests within limits of consent for data sharing.
