# Supplementary material for: Diurnal variation of cardiac autonomic activity in adolescent non-suicidal self-injury
Source: Eur Arch Psychiatry Clin Neurosci. 2023 Mar 5;274(3):609–28. doi: 10.1007/s00406-023-01574-1 (PMC10995014; doi:10.1007/s00406-023-01574-1)
Supplement: Supplementary file 1 — Supplementary file1 (DOCX 164 KB) [file 406_2023_1574_MOESM1_ESM.docx]

**Supplementary Materials**


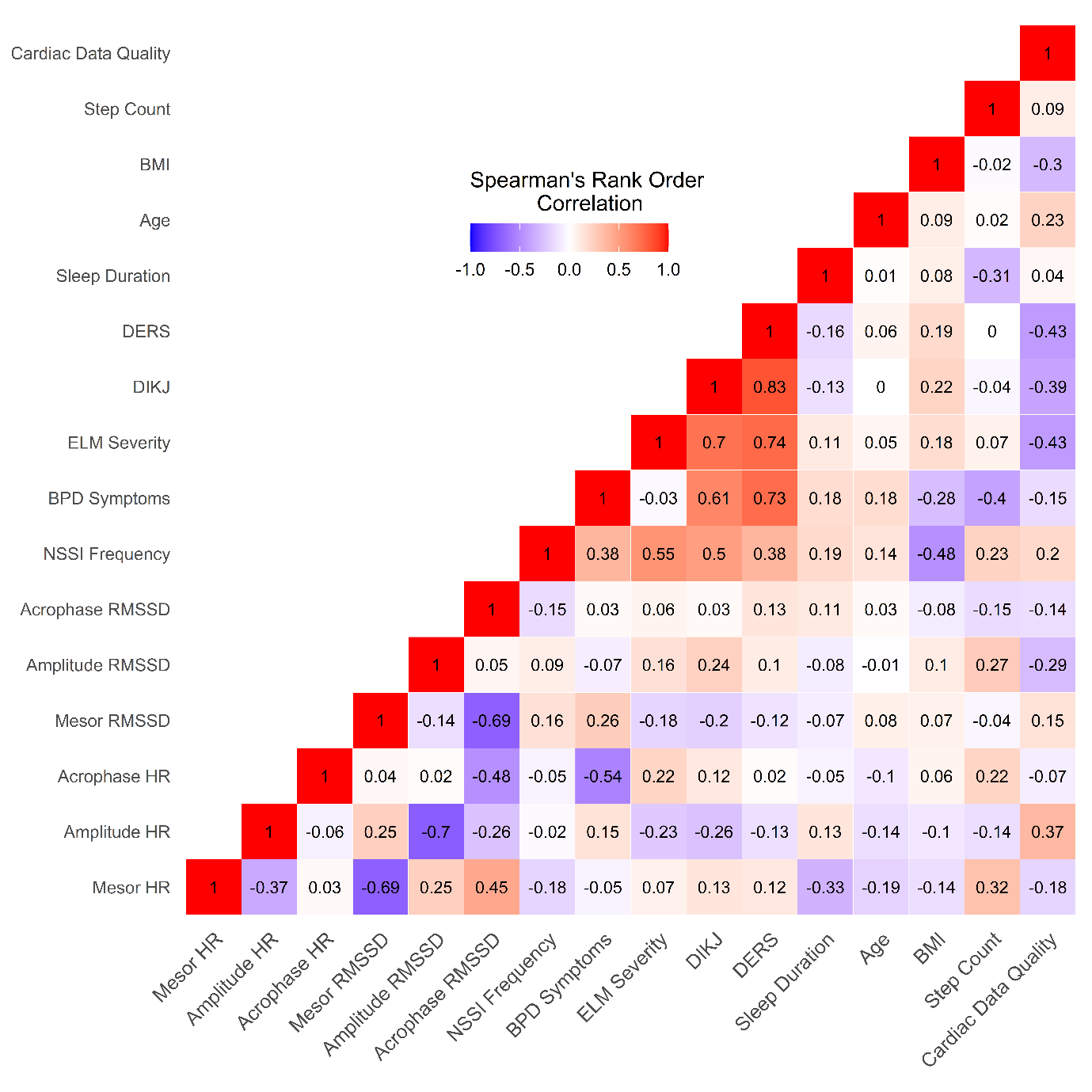


## *Supplementary Figure.* Heat map of Spearman’s Rank Order Correlation Coefficients (Spearman’s ρ) between cosinor parameters of HR and vmHRV, clinical predictors, and potential confounders.

*Supplementary Table 1.* Summary statistics of single (individual-level) cosinor parameters of HR and vmHRV by study group.

| **HR** | | NSSI (*N* = 30) | | | Controls (*N* = 30) | | |
| --- | --- | --- | --- | --- | --- | --- | --- |
|  |  | *N* | M (SD) | Min – Max | *N* | M (SD) | Min - Max |
|  | MESOR | 29 | 85.98 (11.14) | 68.97 – 119.66 | 30 | 79.44 (8.83) | 54.24 – 96.10 |
|  | Amplitude | 29 | -2.91 (5.74) | -15.11 – 7.47 | 30 | .17 (5.05) | -10.55 – 8.18 |
|  | Acrophase | 29 | 12.07 (8.85) | -15.57 – 23.57 | 30 | 12.30 (5.02) | 4.68 – 26.06 |
|  |  |  | | |  | | |
|  |  | NSSI (*N* = 30) | | | Controls (*N* = 30) | | |
| **vmHRV** | | *N* | M (SD) | Min – Max | *N* | M (SD) | Min - Max |
|  | MESOR | 29 | 44.82 (19.55) | 12.16 – 97.17 | 30 | 55.79 (19.77) | 32.46 – 97.15 |
|  | Amplitude | 29 | 4.20 (9.78) | -9.30 – 34.61 | 30 | .28 (6.02) | -9.94 – 15.19 |
|  | Acrophase | 29 | -13.25 (16.27) | -47.37 – 22.13 | 30 | -14.45 (11.13) | -36.73 – 4.58 |

*Note*. HR = Heart rate (bpm); vmHRV = vagally-mediated Heart rate variability (RMSSD, ms). MESOR = Midline estimating statistic of rhythm.

*Supplementary Table 2.*  Fully adjusted multivariate multiple linear regression model results for significant clinical predictors of CVP of ANS activity. The cosinor parameters MESOR and Amplitude of HR were significantly influenced by the variables study group and BPD symptomatology, respectively.

|  | **HR** | | | | | | | | | | | | | | | | |
| --- | --- | --- | --- | --- | --- | --- | --- | --- | --- | --- | --- | --- | --- | --- | --- | --- | --- |
|  | |  | *N* | *B* | 95%[CI] | Std. | *z* | *p* |  |  |  | *N* | *B* | 95%[CI] | Std. | z | *p* |
|  | |  | 59 |  |  |  |  |  |  | |  | 29 |  |  |  |  |  |
| MESOR | | **Study Group** |  | **.57** | **.11 – 1.04** | **.24** | **2.41** | **.016** | MESOR | | BPD |  | -.12 | -.51 – .26 | .20 | -.63 | .529 |
|  |  | **No.  Segments** |  | **-.33** | **-.57 – -.10** | **.12** | **-2.75** | **.006** |  |  | **No.  Segments** |  | **-.47** | **-.77 – -.17** | **.15** | **-3.03** | **.002** |
|  |  | Step count |  | .16 | -.06 - .39 | .11 | 1.45 | .147 |  |  | Step Count |  | .11 | -.17 – .39 | .14 | .75 | .451 |
|  |  | Age |  | -.13 | -.35 - .09 | .11 | -1.20 | .232 |  |  | Age |  | -.36 | -.75 – .03 | .20 | -1.82 | .069 |
|  |  | **BMI** |  | **-.42** | **-.71 - -.14** | **.15** | **-2.90** | **.004** |  |  | **BMI** |  | **-.45** | **-.79 – -.11** | **.17** | **-2.58** | **.010** |
| Amplitude | | Study Group |  | -.32 | -.85 – .21 | .27 | -1.21 | .233 | Amplitude | | **BPD** |  | **.43** | **.06 – .81** | **.19** | **2.26** | **.024** |
|  |  | **No.  Segments** |  | **.32** | **.07 - .58** | **.13** | **2.52** | **.012** |  |  | **No.  Segments** |  | **.51** | **.22 – .81** | **.15** | **3.43** | **.001** |
|  |  | Step count |  | -.13 | -.37 **–** .01 | .12 | -1.12 | .264 |  |  | Step count |  | -.05 | -.32 – .22 | .14 | -.35 | .726 |
|  |  | **Age** |  | **-.26** | **-.49 – -.03** | **.12** | **-2.21** | **.027** |  |  | **Age** |  | **-.42** | **-.80 – -.05** | **.19** | **-2.20** | **.028** |
|  |  | BMI |  | .07 | -.23 **–** .37 | .15 | .45 | .652 |  |  | BMI |  | .13 | -.20 – .46 | .17 | .77 | .440 |
| Acrophase | | Study Group |  | .15 | -.39 – -69 | .27 | .55 | .587 | Acrophase | | BPD |  | -.19 | -.63 – .25 | .22 | -.84 | .401 |
|  |  | **No.  Segments** |  | **.45** | **.19 – .71** | **.13** | **3.45** | **.001** |  |  | **No.  Segments** |  | **.63** | **.29 – .98** | **.18** | **3.61** | **<.001** |
|  |  | Step count |  | .18 | -.05 **–** .42 | .12 | 1.51 | .130 |  |  | Step count |  | .12 | -.20 – .44 | .16 | .75 | .453 |
|  |  | Age |  | -.24 | -.37 **–** .10 | .12 | -1.16 | .247 |  |  | Age |  | -.20 | -.64 – .25 | .23 | -.87 | .386 |
|  |  | BMI |  | .24 | -.07 **–** .54 | .16 | 1.51 | 1.31 |  |  | BMI |  | .32 | -.07 – .70 | .20 | 1.60 | .110 |

*Note.* No. segments = Number of 5-min segments of cardiac data available per participant. Standardized beta-coefficients are reported. MESOR = Midline estimating statistic of rhythm.
